# Breast Cancer Risk From Modifiable and Non-Modifiable Risk Factors among Palestinian Women: A Systematic Review and Meta-Analysis

**DOI:** 10.31557/APJCP.2021.22.7.1987

**Published:** 2021-07

**Authors:** Heba Mohammed Arafat, Julia Omar, Rosediani Muhamad, Tengku Ahmad Damitri Al-Astani, Noorazliyana Shafii, Nahed Ali Al Laham, Ihab Naser, Ohood Mohammed Shamallakh, Kholoud Mohammed Shamallakh, Majed Abed Al Rahman Jebril

**Affiliations:** 1 *Department of Chemical Pathology, School of Medical Sciences, Health Campus, University Sains Malaysia, Kubang Kerian, Kelantan, Malaysia. *; 2 *Department of Family Medicine, School of Medical Sciences, Health Campus, University Sains Malaysia, Kubang Kerian, Kelantan, Malaysia. *; 3 *Department of Laboratory Medicine, Al Azhar University-Gaza, Gaza Strip, Palestine. *; 4 *Department of Clinical Nutrition, Faculty of Applied Medical Sciences, Al- Azhar University- Gaza, Gaza City, Palestine. *; 5 *Department of a Medical Laboratory Sciences, Faculty of Health Sciences, Islamic University of Gaza, Gaza City, Palestine. *; 6 *Global Health Institute, School of Public Health, Xi’an Jiaotong University, Health Science Center, Xi’an, Shaanxi, China. *

**Keywords:** Risk factors, breast cancer, Palestinian women, meta-analysis

## Abstract

**Objective:**

Breast cancer (BC) is known as one of the deadliest forms of cancer, and it is increasing globally. Identifying risk factors for BC is a key point in developing preventive strategies to reduce its occurrence. Herein, we aimed to conduct a systematic review and meta-analysis focus on the risk factors for BC in Palestine.

**Material and Methods:**

We performed a systematic search via PubMed, MEDLINE, SCOPUS, Science Direct, Cochrane library, Emerald Insight, and Google scholar for identifying studies published on BC risk factors up to March 2021. Pooled odds ratios (OR) are calculated using fixed and random-effect models. Data were processed using Review Manager 5.4 (RevMan 5.4).

**Results:**

From a total of 73 articles, seven case-control studies met the criteria for systematic review. Meta-analysis results showed that of the known modifiable risk factors for BC, diabetes mellitus (DM) had the highest odds ratio (OR = 4.97, 95% CI 3.00- 8.25) followed by hypertension (OR = 3.21, 95% CI 1.96-5.23), obesity (BMI >30 Kg/m^2^) (OR = 2.90, 95% CI 2.00- 4.21), and passive smoking (OR = 1.50, 95% CI 1.12- 2.02). Controversially, breastfeeding (OR = 0.37, 95% CI 0.23- 0.61) was protective factor in BC. Of non-modifiable risk factors for BC has reached menopause had the highest odds ratio (OR = 3.74, 95% CI 2.64- 5.29), followed by family history of BC (OR = 2.63, 95% CI 1.07-6.44) and age (≥ 40 years) (OR = 2.49, 95% CI 1.43-4.34).

**Conclusions:**

The most significant predictors of BC in Palestine were DM, hypertension, passive smokers, age (>40), reached menopause, and family history of BC. Almost all these risk factors are consistent with known risk factors for breast cancer in other parts of the world.

## Introduction

Cancer is the second leading cause of death globally and is responsible for an estimated 9.6 million deaths in 2018 (World Health Organization, 2018). Breast cancer (BC) is the most diagnosed cancer among women worldwide, accounting for 1 in 4 cancer cases. It is the most frequent cancer amongst both sexes and is the leading cause of death from cancer in women. The estimated 2.3 million new cases indicated that one in every 8 cancers diagnosed in 2020 was BC. In 2020, there were an estimated 684,996 deaths from BC, with a disproportionate number of these deaths occurring in low-resource settings (World Health Organization, 2020).

The cause of BC is multifactorial. Several risk factors for BC have been known for some time. The risk factors are differentiated into non-modifiable risk factors: such as sex, age, genetic characteristics including family or personal history of BC, ethnicity, and early menarche or menopause. Modifiable risk factors, usually associated with lifestyle factors, include alcohol consumption, excess weight or obesity, physical inactivity, parity, and use of some medications, such as oral contraceptives (Youn and Han, 2020).

The incidence of BC has been increasing in Palestine in recent years, partly due to growing awareness and detection and the lifestyle and dietary habits related to poverty. It is the most prevalent cancer among Palestinian women, accounting for 32% of cancer diagnoses in the West Bank and 18% of those in the Gaza Strip. BC is most treatable when detected early. Unfortunately, more than 60% of BC cases in Palestine are found at a late stage, reducing the chance of survival (United Nation Population Fund, 2020).

Breast cancer was the third largest cause of cancer mortality in 2018 in the occupied Palestinian territories (oPt) at 12%, after lung cancer (20%) and colon cancer (13%) (Ministry of Health, 2018). The 5-year survival rate after the diagnosis of BC is considerably worse than in countries with strong healthcare systems. Diagnostic facilities in Gaza are scarce. There is no clear evidence to assess the quality, cost-effectiveness, and accuracy of pathological services in Gaza; carrying out immunohistochemistry testing is complicated by the inadequate supply of slides and reagents, and limited human resources cause appointment delays (AlWaheidi, 2019). Surgery and chemotherapy, but not radiation therapy, is generally available. There is no comprehensive cancer center offering all treatment modalities and services and there are few support services, such as palliative care, nutrition, psychosocial support, or rehabilitation (Gale et al., 2018).

There is however limited data on BC risk factors among the Palestinian population. The primary objectives of this study were to conduct a systematic literature review and meta-analysis on the risk factors of BC during the last decade among Palestinian women and summarize the findings of these studies.

## Materials and Methods

*Protocol and Registration*

This systematic review and meta-analysis followed the Preferred Reporting Items for Systematic Reviews and Meta-analyses (PRISMA) checklist. The protocol was registered in the PROSPERO international prospective register of systematic reviews (PROSPERO registration number: (CRD42021236450)).

*Data Sources*

Online international databases (PubMed, MEDLINE, SCOPUS, Science Direct, Cochrane library, Emerald Insight, and Google scholar) searched for studies reporting primary data on the epidemiology of BC among Palestinian women from inception to 10 March 2021.

*Search Strategy*

Controlled vocabulary terms were appropriately incorporated for each database. The search terms were MeSH terms and text words linked to risk factors and BC, using a combination of the following search terms: risk factors, causes, breast cancer, breast neoplasm, breast tumor, women, Palestine, West Bank, Gaza Strip, Occupied Palestinian Territory. The search strategy was tested in two databases (PubMed, Google Scholar) and was further refined based on its ability to retrieve known relevant studies according to each database. Forward and backward reference chaining of included studies were carried out in which the reference lists from the included papers were searched to identify other relevant information. A systematic literature search of multiple databases using search terms as listed above.

*Study Eligibility*

The investigator independently screened all titles and abstracts from the initial search results and full-text articles identified from the first-stage screening (titles and abstract). Searches were conducted in English. Any observational (cross-sectional, cohort, longitudinal, and case-control) studies were eligible for inclusion if the study reported the target population of interest (BC patients) and on study outcomes (epidemiology, prevalence or frequency, and risk factors of BC). Also, we will consider Clinical trials – both randomized and non – randomized that have data on BC risk factors. Studies published in the English language in the last decade from 1st Jan 2010 to 31st Dec 2020 in Palestine were included. Studies of conference proceedings, case reports, qualitative studies, opinion papers, letters, only abstracts, book chapters, editorials, and review articles were excluded. Studies with the primary objective of examining the correlation of BC to other types of cancers, insufficient data, and irrelevant outcomes were excluded.

*Data Extraction*

Data Extraction Search results from each database were downloaded in a standardized tag format developed by Research Information Systems (.ris) or NBIB format (.nbib). In databases that do not allow all search results to be downloaded at once (e.g., Google Scholar), search results were downloaded in sections and later merged in the EndNote library and duplicates were removed. After removing duplicates, the search result was exported as Text File (*.txt) format and later converted to Excel workbook (.xlsx) format. Preliminary screening of titles and abstracts was conducted to identify potential articles of interest. The full texts of potentially eligible studies were retrieved and re-assessed for inclusion/exclusion criteria. Assessment of eligibility was made in duplicate and independently, to avoid bias in study selection. Following the full-text review, a detailed assessment of why studies were excluded was prepared. After study identification, data from included studies were abstracted using a standardized pre-design and pre-piloted electronic data abstraction form, in Microsoft Excel format, to assess study quality and for evidence synthesis. Data abstractions were conducted independently to minimize the risk of errors. The information abstracted included: author’s name, publication year, region, number of participants, method of data collection, and risk factors. When there were multiple publications of the same study, data were extracted from each publication, but only the most “complete” and up-to-date data were included. The data were analyzed following the resolution of overlaps in the extracted data. The literature search and screening output were reported in a Preferred Reporting Items for Systematic Reviews and Meta-Analyses (PRISMA) study flow diagram.

*Quality Assessment*

All retrieved papers included in the review underwent a quality assessment process conducted by two independent reviewers independently and in duplicate, using a standardized critical appraisal quality assessment tool. All disagreement was resolved by discussion with the involvement of a third review author.

*Statistical Analysis*

The qualitative synthesis omitted studies with a high risk of bias. Aggregate level data was used for data synthesis, and a summary of all the findings in the included studies was provided. The Cochrane’s Q test was performed to evaluate the heterogeneity among the included retrospective studies, and the I^2^ statistic was also calculated. Significant heterogeneity was considered if I^2^ > 50%. A random-effect model was used to pool the results if significant heterogeneity was found; otherwise, a fixed-effect model was applied. The RevMan (Version 5.4.1; Cochrane Collaboration, Oxford, UK) was used for the statistics.

## Results

*Study Selection and Characteristics*

**Figure 1:**
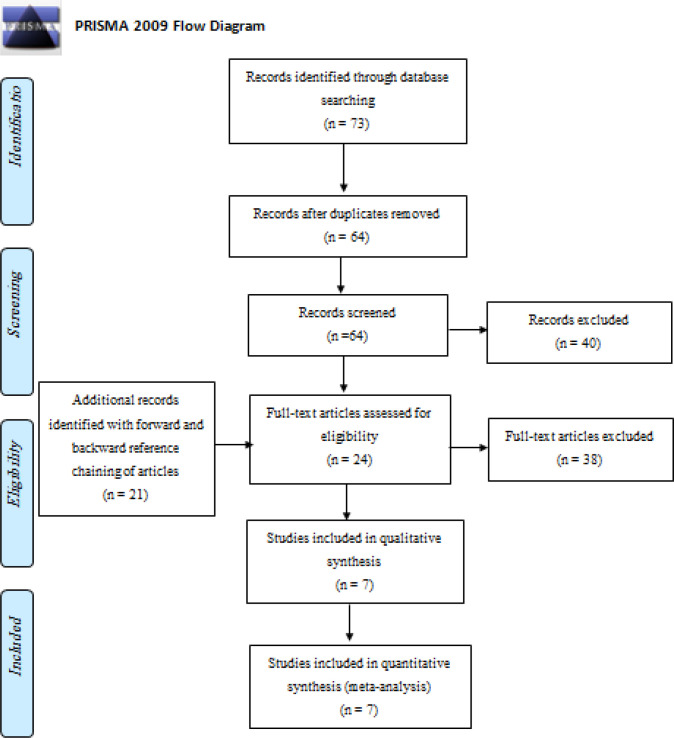
Preferred Reporting Items for Systematic Reviews and Meta-Analyses (The PRISMA) Flowchart

Our initial search through databases resulted in 73 papers. After excluding duplicated papers, 64 papers were screened by their titles and abstracts, from which 24 articles qualified for a full-text review. Forward and backward reference chaining of articles during full-text review identified 21 extra articles. In total, 45 articles were assessed for eligibility in full text, and from these, 7 related to BC risk from modifiable and non-modifiable risk factors among women in Palestine between 2010- 2020 (Figure1: PRISMA Flow of information diagram). 

*Study characteristics*

All the seven included studies were case-control. The studies’ sample size ranged from 150 to 474, including 2032 participants. Six studies were conducted in the Gaza Strip, and one in the West Bank. Characteristics of studies entered into meta-analysis presented in Table 1. 

*Risk factors for breast cancer*

**Figure 2:**
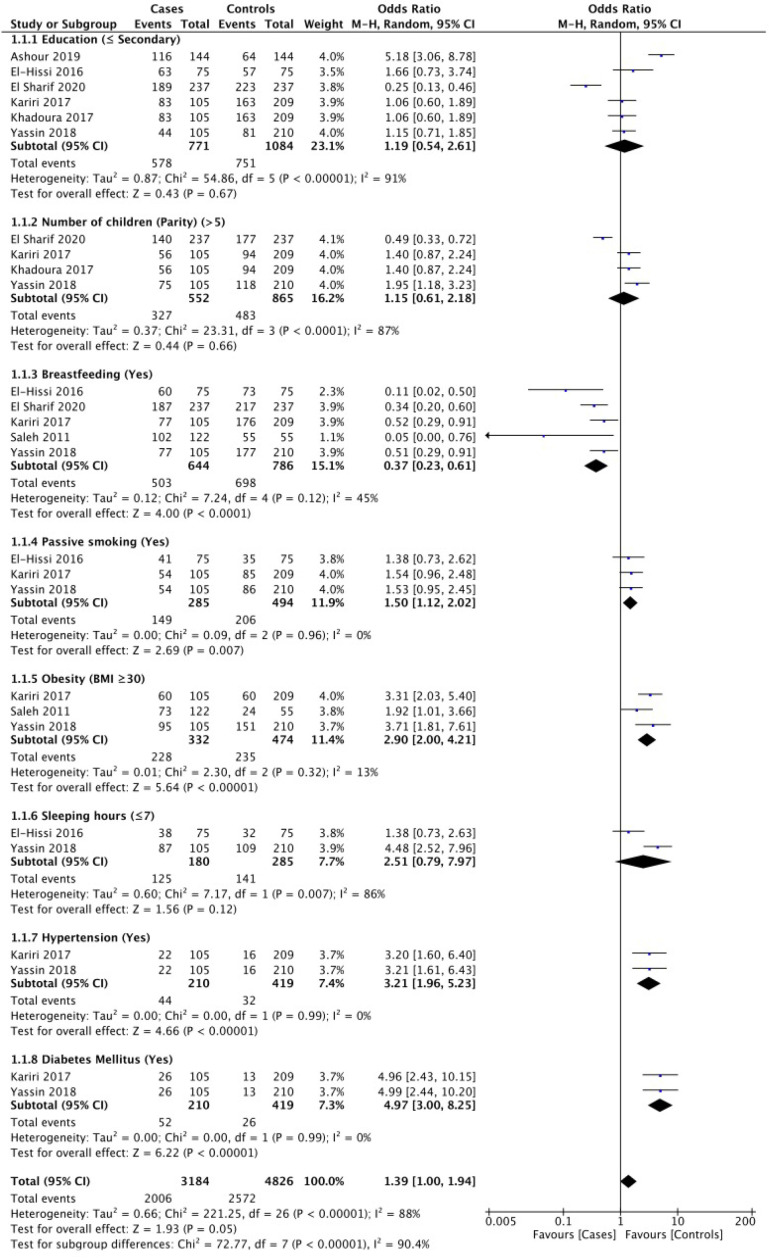
Modifiable Breast Cancer Risk Factors

**Figure 3:**
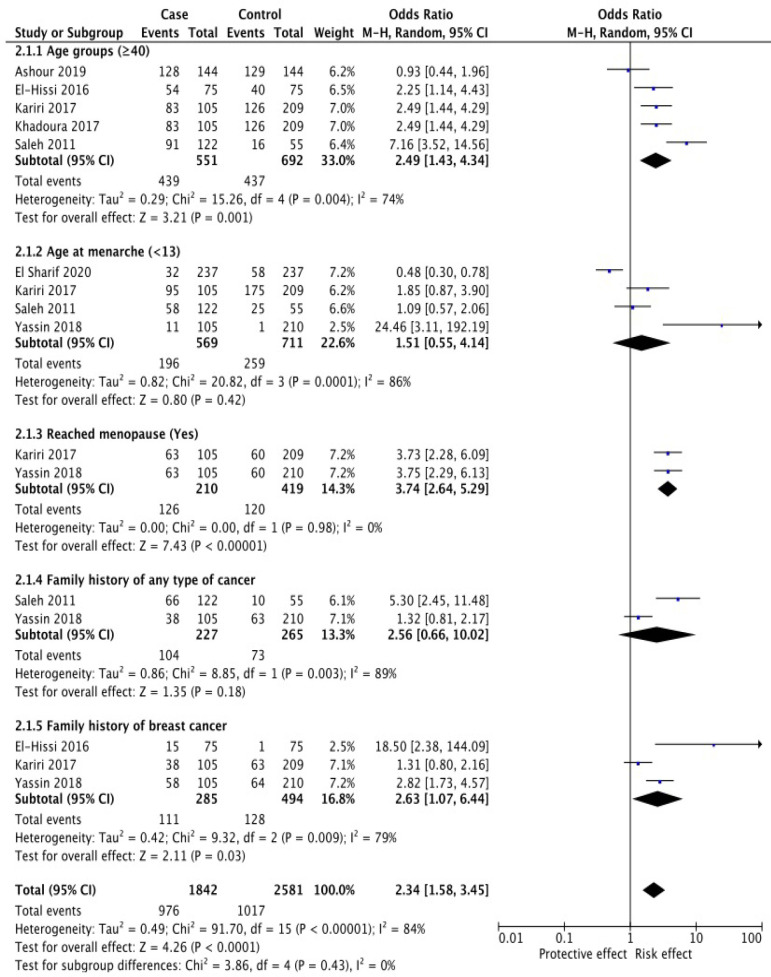
Non-Modifiable Breast Cancer Risk Factors

Based on the results of the systematic review, there were seven case-control studies analyzed by meta-analysis. The research variables analyzed based on the systematic review that has been done were modifiable risk factors including education, breastfeeding, parity, obesity, passive smoking, sleeping hours, hypertension, and diabetes (Figure 2). Non-modifiable risk factors including age groups (years), age at menarche, reached menopause, family history of any type of cancer, and family history of BC (Figure 3).

Figure 2 based on modifiable BC risk factors known diabetes mellitus (DM) had the highest odd ratio (OR = 4.97, 95% CI 3.00- 8.25) followed by hypertension (OR = 3.21, 95% CI 1.96-5.23), obesity (BMI >30 Kg/m^2^) (OR = 2.90, 95% CI 2.00- 4.21), and passive smoking (OR = 1.50, 95% CI 1.12- 2.02)., Controversially, breastfeeding (OR = 0.37, 95% CI 0.23- 0.61) was protective factor of BC. While sleeping hours (<7 hours), education (< secondary), and parity (>5 children) were not associated with BC. 

Figure 3 based on non-modifiable risk factors for BC reached menopause had the highest odd ratio (OR = 3.74, 95% CI 2.64- 5.29), followed by family history of BC (OR = 2.63, 95% CI 1.07-6.44) and age (≥ 40 years) (OR = 2.49, 95% CI 1.43-4.34). 

*Individual-related risk factors (* Table 2 *)*

*Modifiable Breast Cancer Risk Factors*

*Education level*

This factor was studied in six articles. According to meta-analysis, no significant differences were found for both who are less than secondary level (OR: 1.19, 95%CI 0.54, 2.61) and more than secondary level (Figure 2). 

*Number of children (Parity)*

Four studies were included with this factor. According to meta-analysis, no significant differences were found for both having more than five children (OR: 1.15, 95%CI 0.61–2.18) and having less than five children (Figure 2). 

*Breast feeding*

This factor was investigated in five studies. Breastfeeding showed a protective effect on the occurrence of BC (OR: 0.37, 95%CI 0.23–0.61). No significant heterogeneity was observed (I^2^ = 45%, P = 0.12) (Figure 2). 

*Passive smoking*

Three papers studied this factor. The meta-analysis showed that the odds of BC occurrence were 1.50 times higher in the passive smokers (OR: 1.50, 95%CI 1.12–2.02). No significant heterogeneity was observed (I^2^ = 0%, P = 0.96) (Figure 2). 

*Obesity (BMI >30 Kg/m* ^2^ *)*

BMI was investigated in three studies. The meta-analysis indicated that the odds of BC occurrence were 2.90 times higher in the group with obesity (BMI >30) (OR: 2.90, 95%CI 2.00–4.21). No significant heterogeneity was observed (I^2^ = 13%, P = 0.32) (Figure 2).

*Sleeping hours*

This factor was investigated in two studies. The meta-analysis showed no significant difference between groups for BC occurrence regarding sleeping hours (OR: 2.51, 95%CI 0.79–7.97). A significant heterogeneity was observed (I^2^ = 86%, P = 0.007) (Figure 2).

*Hypertension*

Two studies reported on hypertension. The meta-analysis between the two groups showed that the odds of BC development was 3.21 times higher in subjects with hypertension (OR: 3.21, 95%CI 1.96–5.23). No significant heterogeneity was observed (I^2^ = 0%, P = 0.99) (Figure 2).

*Diabetes mellitus*

This factor was investigated in two studies. The meta-analysis between the two groups showed that the odds of BC development was 4.97 times higher in subjects with DM than non-diabetic patients (OR: 4.97, 95%CI 3.00–8.25). No significant heterogeneity was observed (I^2^=0%, P = 0.99) (Figure 2). 

*Non-Modifiable Breast Cancer Risk Factors*

*Age*

Six papers studied this factor. The meta-analysis showed that the odds of BC occurrence were 2.49 times higher in the age ≥ 40 years (OR: 2.49, 95%CI 1.43–4.34). A significant heterogeneity was observed (I^2^ = 74%, P = 0.004) (Figure 3).

*Age at Menarche*

This factor was studied in four articles. The meta-analysis showed no significant difference between groups for BC occurrence regarding age at menarche <13 (OR: 1.51, 95%CI 0.55–4.14). Significant heterogeneity was observed in this regard (I^2^ = 86%, P = 0.0001) (Figure 3).

*Reached Menopause*

Two studies have investigated this factor. A significant relationship was observed between groups in this regard (OR: 3.74, 95%CI 2.64–5.29). No heterogeneity was observed (I^2^ = 0%, P = 0.98) (Figure 3).

*Family history of any type of cancer*

Two studies reported a family history of BC. The meta-analysis between the two groups showed that the odds of BC development were not significant in subjects with a family history of any type of cancer (OR: 2.56, 95%CI 0.66–10.02). High heterogeneity was observed (I^2^ = 89%, P = 0.003) (Figure 3).

*Family history of breast cancer*

Three studies reported a family history of BC. The meta-analysis between the two groups showed that the odds of BC development were 2.63 times higher in subjects with a family history of BC (OR: 2.63, 95%CI 1.07–6.44). High heterogeneity was observed (I^2^ = 79%, P = 0.009) (Figure 3).

**Table 1:** Characteristic of Included Studies to Assess Risk Factors of Breast Cancer in Palestine

Author, (year)	Region	No. of participants (Case/Control)	Method of data collection	Risk factors
Ashour, A., & Safi, J., (2011) (Ashour, 2011)	Gaza Strip	288; (144/144)	Face to face interviewed questionnaire	- Educational status, and age.
El Sharif, N., & Khatib, I., (2020) (El Sharif and Khatib, 2020)	West Bank	474; (237/ 237)	Structured questionnaire	- Age at menarche, education, parity, and breastfeeding.
Kariri et al., (2017) (Kariri et al., 2017)	Gaza Strip	314; (105/209)	Face-to-face interviewed questionnaire	- Obesity, family history of BC, hypertension, education, number of children, breastfeeding, passive smoking, diabetes, age, age at menarche, and reached menopause.
Khadoura, K. J., (2017) (Khadoura, 2017)	Gaza Strip	314; (105/209)	Face to face questionnaire	- Education, number of children, and age.
Saleh, M. S., (2011) (Saleh, 2011)	Gaza Strip	177; (122/55)	Face to face interviewed questionnaire	- Age, breastfeeding, family history of any type of cancer, obesity, and age at menarche.
Yassin et al., (2018) (Yassin, 2018)	Gaza Strip	315; (105/210)	Structured questionnaire	- Obesity, family history of any type of cancer, hypertension, education, number of children, breastfeeding, passive smoking, diabetes, age, age at menarche, reached menopause, sleeping hours, and family history of BC.
El-Hissi et al., (2016) El-Hissi et al., (2016)	Gaza Strip	150; (75/75)	Semi-structured face-to-face interviews	- Education, breastfeeding, passive smoking, sleeping hours, age, and family history of BC.

**Table 2:** Pooled Estimation of ORs Obtained from Meta-Analysis of Risk Factors of Breast cancer in Palestine

Risk factors	Included studies	Pooled OR	95% CI
Modifiable Breast Cancer Risk Factors			
Education (< secondary)	6	1.19	0.54- 2.61
Number of children (Parity) (>5 children)	4	1.15	0.61- 2.18
Breastfeeding (yes)	5	0.37	0.23- 0.61
Passive smoking (yes)	3	1.5	1.12- 2.02
Obesity (BMI >30)	3	2.9	2.00- 4.21
Sleeping hours (<7 hours)	2	2.51	0.79- 7.97
Hypertension	2	3.21	1.96- 5.23
Diabetes mellitus	2	4.97	3.00- 8.25
Non-Modifiable Breast Cancer Risk Factors			
Age groups (≥40 years)	6	2.49	1.43- 4.34
Age at menarche (<13)	4	1.51	0.55- 4.14
Reached menopause (yes)	2	3.74	2.64- 5.29
Family history of any type of cancer	2	2.56	0.66- 10.02
Family history of BC	3	2.63	1.07- 6.44

## Discussion

We undertook this systematic review and meta-analysis to identify the risk factors contributing to the occurrence of female BC in Palestine. Out of 7 included papers, more than 21 factors were studied as BC risk factors, of which only 13 factors entered the meta-analysis. Out of all risk factors, factors including, obesity, passive smoking, hypertension, diabetes, age groups (years), reached menopause, and family history of BC were significantly associated with a higher chance of BC development. In contrast, breastfeeding was demonstrated to be protective. The other remaining risk factors were not associated with the development of BC. 

In our meta-analysis, passive smokers were at higher risk of BC development. Our findings were in the same line with the systematic review of Shamshirian et al. (2020) among Iranian females, which implied that passive smokers were at higher risk of BC development (OR: 1.84, 95%CI 1.43–2.25) (Shamshirian et al., 2020). Moreover, some other studies reported a significant association between passive smoking and the risk of BC (Macacu et al., 2015; Regev-Avraham et al., 2018). Smoking has been linked to an underlying tumor progression mechanism, and increased epithelial-to-mesenchymal transition and motility have been observed in breast tumor cells after they have been exposed to cigarette smoke (Jones et al., 2017).

Regarding obesity (BMI >30 Kg/m^2^), our results were in the same line with meta-analysis in the Eastern Mediterranean Region, which indicated the increased risk of BC in individuals with BMI 25-30 Kg/m^2^, and BMI >30 Kg/m^2^ (Namiranian et al., 2014). These findings are also supported by individual studies (Saleh et al., 2008; Awatef et al., 2010). Obesity has been shown to trigger a reduction in sex hormone-binding globulin (SHBG) and consequentially an increase in the bioavailability of estrogen. Insulin resistance is common in obese women and is associated with hyperinsulinaemia. Insulin can stimulate mammary epithelium in vitro via the effects of insulin like growth factor 1(IGF 1) which has a synergistic effect with estrogen in promoting mammary carcinogenesis (Ballard-Barbash et al., 1990; Carmichael and Bates, 2004).

Diabetes Mellitus was one of the associated risk factors for BC development in our study; the odds of BC development were nearly 5 times higher in subjects with DM than non-diabetic patients. (OR: 4.97, 95%CI 3.00–8.25). Our result is inconsistent with the meta-analysis done by Larsson et al. (2007) showed that women with (versus without) diabetes had a statistically significant 20% increased risk of BC (RR, 1.20; 95% CI, 1.12–1.28) (Larsson et al., 2007). Besides, the study of Anothaisintawee et al. demonstrated that DM was a risk factor for BC with the pooled adjusted OR of 1.14 (95% CI = 1.09- 1.19) (Anothaisintawee et al., 2013). The explanation of the association between DM and the risk of BC is that insulin has been shown to have a mitogenic effect upon BC cells in vitro through several mechanisms. It can act synergistically with estradiol and stimulate the proliferation of the cell line. Insulin can also lower SHBG production, thereby increasing biologically available estradiol (Bhandari et al., 2014). 

Our results suggest a significant association between hypertension and the risk of BC. In the same direction, meta-analyses carried out by Seretis et al., (2019) suggested that hypertension was associated with a 7% higher risk of total BC. These findings agree with a recent meta-analysis of 12 prospective studies by Han et al., (2017) that reported a 7% higher risk for total breast risk in hypertensive individuals. Suggestive mechanisms to explain this association involve blocking apoptosis, adipose tissue-related hypoxia, and chronic inflammation promoting reactive oxygen species formation (Rausch et al., 2017).

We found that breastfeeding plays a protective role against BC development. Our findings were in the same direction with numerous meta-analyses carried out by Islami et al., (2015), Unar-Munguía et al., (2017), and González-Jiménez (2018) in which their meta analysis justifies that breastfeeding led to a significant reductions in the risk of developing BC (Islami et al., 2015; Unar-Munguía et al., 2017; González-Jiménez, 2018). Additionally, other studies support the inverse association between breastfeeding and BC risk (Awatef et al., 2010).

Several mechanisms have been proposed to explain the observed association these include the following: (1) reduced exposure to the cyclic hormones of reproductive life due to the ovulatory suppression that occurrs with prolonged breastfeeding; (2) a protective effect from direct physical changes in the breast that accompany milk production; and (3) a reduction in the concentrations of toxic organochlorines in the breast with increasing cumulative duration of breastfeeding (Zhou et al., 2015). 

In our meta-analysis, we found that older age can increase the chance of BC development (OR = 2.49, 95% CI 1.43-4.34). This finding agreed with the meta-analysis done by Nindrea et al., (2017) that confirmed the associations between age (≥ 40 years) and the risk of BC (Nindrea et al., 2017). Moreover, some other studies indicated a significant association between older age and the risk of BC (Yip and Lau, 2008; Ekpanyaskul et al., 2010; Sangrajrang et al., 2013).

Based on the results of the meta-analysis, women who have reached menopause have around four times higher risk of BC occurrence compared to those who have not reached menopause (OR: 3.74, 95%CI 2.64–5.29). Similarly, the Collaborative Group on Hormonal Factors in BC meta-analysis also reported the same conclusions (Collaborative Group on Hormonal Factors in Breast, 2012). In addition, a study done by Sangrajrang et al., (2013) has found a significant positive association with an increased risk of BC observed in postmenopausal women.

A family history of BC was one of the associated risk factors for BC development in our study. In one of the first meta-analysis on “Family history and the risk of BC”, Pharoah et al., (1997) pooled estimate of relative risk (RR) indicated that the probability of BC occurrence is higher in those individuals with a family history of this malignancy (RR: 1.9, 95%CI, 1.7–2.0) (Pharoah et al., 1997). Many other studies reported the association of family history with the risk of BC (Brewer et al., 2017; Nindrea et al., 2017; Shamshirian et al., 2020). Women with a family history of BC should be counseled and educated about the risk of BC, and it is recommended to provide for early detection of BC. 

In conclusion, based on this systematic review and meta-analysis, factors including DM, hypertension, passive smoking, age (≥40), have reached menopause, and family history of BC, all play a significant role in the development of BC. In contrast, breastfeeding showed a significant inverse association with BC occurrence. This systematic review and meta-analysis highlighted the current gaps in our knowledge and stressed the importance of further investigations needed to improve BC risk management in BC survivors.

## Author Contribution Statement

Conceptualization, H.M.A., J.O., O.M.SH., N.S., I.A.N., N.A.L., KH.M.SH., M.A.J., R.B.M., and T.A.D.A.; Methodology, H.M.A., J.O., O.M.SH., and N.S.; Validation, H.M.A., J.O., and O.M.SH.; Formal analysis, H.M.A., and O.M.SH; Data curation, H.M.A., J.O., O.M.SH., and N.S.; Writing—original draft preparation, H.M.A., and O.M.SH.; Writing—review and editing, H.M.A., J.O., O.M.SH., N.S., I.A.N., N.A.L., KH.M.SH., M.A.J., R.B.M., and T.A.D.A. All authors have read and agreed to the published version of the manuscript.

## Data Availability

The datasets analyzed during the current study are available from the corresponding or first author on reasonable request.

## Approval

The study includes secondery data analysis, therfore dosen’t require approval from scientific body. This systematic review on breast cancer risk from modifiable and non-modifiable risk factors among palestinian women is a part of the dissertation submitted in fulfillment of the requirements for the degree of Doctor of Philosophy (Cancer biolgy, Chemical pathology department, Universiti Sains Malaysia, Malaysia).

## Registration

This systematic review and meta analysis was registered in the PROSPERO international prospective register of systematic reviews (PROSPERO registration number: (CRD42021236450)).

## References

[B1] AlWaheidi S (2019). Breast cancer in Gaza—a public health priority in search of reliable data. Ecancermedicalscience.

[B2] Anothaisintawee T, Wiratkapun C, Lerdsitthichai P (2013). Risk factors of breast cancer: a systematic review and meta-analysis. Asia Pac J Public Health.

[B3] Ashour A (2011). Environmental Risk Factors Associated with Breast Cancer-Gaza Governorates.

[B4] Awatef M, Olfa G, Imed H (2010). Breastfeeding reduces breast cancer risk: a case–control study in Tunisia. Cancer Causes Control.

[B5] Ballard-Barbash R, Schatzkin A, Taylor PR (1990). Association of change in body mass with breast cancer. Cancer Res.

[B6] Bhandari R, Kelley GA, Hartley TA (2014). Metabolic syndrome is associated with increased breast cancer risk: a systematic review with meta-analysis. Int J Breast Cancer.

[B7] Brewer HR, Jones ME, Schoemaker MJ (2017). Family history and risk of breast cancer: an analysis accounting for family structure. Breast Cancer Res Treat.

[B8] Carmichael AR, Bates T (2004). Obesity and breast cancer: a review of the literature. Breast J.

[B9] Collaborative Group on Hormonal Factors in Breast C (2012). Menarche, menopause, and breast cancer risk: individual participant meta-analysis, including 118 964 women with breast cancer from 117 epidemiological studies. Lancet Oncol.

[B10] Ekpanyaskul C, Khuhaprema T, Wiangnon S (2010). Case-control study of occupational categories and breast cancer risk in Thailand. Asian Pac J Cancer Prev.

[B11] El-Hissi JH, El-Batrokh DM, Al-Masri IM (2016). Nutritional factors associated with breast cancer in Gaza Strip-Palestine: A Hospital Based Study. J Adv Med Pharm Sci.

[B12] El Sharif N, Khatib I (2020). Reproductive factors and breast cancer risk in Palestine: A Case-control Study.

[B13] Gale RP, Halahleh K, Williams P (2018). Cancer Care in the Palestinian Territories. ASCO Post. October.

[B14] González-Jiménez E (2018). Breastfeeding and reduced risk of breast cancer in women: a review of scientific evidence Selected Topics in Breastfeeding, R. IntechOpen.

[B15] Han H, Guo W, Shi W (2017). Hypertension and breast cancer risk: a systematic review and meta-analysis. Sci Rep.

[B16] Islami F, Liu Y, Jemal A (2015). Breastfeeding and breast cancer risk by receptor status—a systematic review and meta-analysis. Ann Oncol.

[B17] Jones ME, Schoemaker MJ, Wright LB (2017). Smoking and risk of breast cancer in the Generations Study cohort. Breast Cancer Res.

[B18] Kariri M, Jalambo MO, Kanou B (2017). Risk factors for breast cancer in Gaza Strip, Palestine: A case-control study. Clin Nutr Res.

[B19] Khadoura KJ (2017). socio demographic risk factors associated with breast cancer in gaza strip. J Nurs Womens Health.

[B20] Larsson SC, Mantzoros CS, Wolk A (2007). Diabetes mellitus and risk of breast cancer: a meta-analysis. Int J Cancer.

[B21] Macacu A, Autier P, Boniol M (2015). Active and passive smoking and risk of breast cancer: a meta-analysis. Breast Cancer Res Treat.

[B23] Namiranian N, Moradi-Lakeh M, Razavi-Ratki SK (2014). Risk factors of breast cancer in the Eastern Mediterranean Region: a systematic review and meta-analysis. Asian Pac J Cancer Prev.

[B24] Nindrea RD, Aryandono T, Lazuardi L (2017). Breast cancer risk from modifiable and non-modifiable risk factors among women in Southeast Asia: a meta-analysis. Asian Pac J Cancer Prev.

[B25] Pharoah PD, Day NE, Duffy S (1997). Family history and the risk of breast cancer: a systematic review and meta-analysis. Int J Cancer.

[B26] Rausch LK, Netzer NC, Hoegel J (2017). The linkage between breast cancer, hypoxia, and adipose tissue. Front Oncol.

[B27] Regev-Avraham Z, Baron-Epel O, Hammond S (2018). Passive smoking, NAT2 polymorphism, and breast cancer risk in Israeli Arab women: a case–control study. Breast Cancer.

[B28] Saleh F, Reno W, Ibrahim G (2008). The first pilot study on characteristics and practice patterns of Kuwaiti breast cancer patients. J Environ Pathol Toxicol Oncol.

[B29] Saleh MS (2011). Reproductive factors and common genetic mutations associated with breast cancer risk in Gaza Strip (Doctoral dissertation, Islamic University–Gaza). Asian Pac J Cancer Prev.

[B30] Seretis A, Cividini S, Markozannes G (2019). Association between blood pressure and risk of cancer development: a systematic review and meta-analysis of observational studies. Sci Rep.

[B31] Shamshirian A, Heydari K, Shams Z (2020). Breast cancer risk factors in Iran: a systematic review & meta-analysis. Horm Mol Biol Clin Investig.

[B32] Unar-Munguía M, Torres-Mejia G, Colchero MA (2017). Breastfeeding mode and risk of breast cancer: a dose–response meta-analysis. J Hum Lact.

[B36] Yassin SY, Malak A, Samer D, Maher M, Ayman A (2018). Extrinsic risk factors for women breast cancer in Gaza Strip, Palestine: Associations and Interactions in a Case-Control Study. Adv Breast Cancer Res.

[B37] Yip CH, Lau P-C (2008). Does a positive family history influence the presentation of breast cancer?. Asian Pac J Cancer Prev.

[B38] Youn HJ, Han W (2020). A review of the epidemiology of breast cancer in Asia: Focus on risk factors. Asian Pac J Cancer Prev.

[B39] Zhou Y, Chen J, Li Q (2015). Association between breastfeeding and breast cancer risk: evidence from a meta-analysis. Breastfeed Med.

